# Eradication of *Staphylococcus aureus* Biofilm Infection by Persister Drug Combination

**DOI:** 10.3390/antibiotics11101278

**Published:** 2022-09-20

**Authors:** Rebecca Yee, Yuting Yuan, Andreina Tarff, Cory Brayton, Naina Gour, Jie Feng, Ying Zhang

**Affiliations:** 1 Department of Molecular Microbiology and Immunology, Bloomberg School of Public Health, Johns Hopkins University, Baltimore, MD 21205, USA; 2 Department of Graduate Medical Education, Louis A. Weiss Memorial Hospital, Chicago, IL 60640, USA; 3 Department of Comparative Medicine, Johns Hopkins University School of Medicine, Baltimore, MD 21205, USA; 4 The Solomon H. Snyder Department of Neuroscience, Johns Hopkins University School of Medicine, Baltimore, MD 21205, USA; 5 School of Basic Medical Sciences, Lanzhou University, Lanzhou 730000, China; 6 State Key Laboratory for Diagnosis and Treatment of Infectious Diseases, National Clinical Research Center for Infectious Diseases, National Medical Center for Infectious Diseases, Collaborative Innovation Center for Diagnosis and Treatment of Infectious Diseases, The First Affiliated Hospital, Zhejiang University School of Medicine, Hangzhou 310003, China

**Keywords:** *Staphylococcus aureus*, persisters, biofilm, antimicrobial activity, drug combination

## Abstract

*Staphylococcus aureus* can cause a variety of infections, including persistent biofilm infections, which are difficult to eradicate with current antibiotic treatments. Here, we demonstrate that combining drugs that have robust anti-persister activity, such as clinafloxacin or oritavancin, in combination with drugs that have high activity against growing bacteria, such as vancomycin or meropenem, could completely eradicate *S. aureus* biofilm bacteria in vitro. In contrast, single or two drugs, including the current treatment doxycycline plus rifampin for persistent *S. aureus* infection, failed to kill all biofilm bacteria in vitro. In a chronic persistent skin infection mouse model, we showed that the drug combination clinafloxacin + meropenem + daptomycin which killed all biofilm bacteria in vitro completely eradicated *S. aureus* biofilm infection in mice while the current treatments failed to do so. The complete eradication of biofilm bacteria is attributed to the unique high anti-persister activity of clinafloxacin, which could not be replaced by other fluoroquinolones including moxifloxacin, levofloxacin, or ciprofloxacin. We also compared our persister drug combination with the current approaches for treating persistent infections, including gentamicin + fructose and ADEP4 + rifampin in the *S. aureus* biofilm infection mouse model, and found neither treatment could eradicate the biofilm infection. Our study demonstrates an important treatment principle, the Yin–Yang model, for persistent infections by targeting both growing and non-growing heterogeneous bacterial populations, utilizing persister drugs for the more effective eradication of persistent and biofilm infections. Our findings have implications for the improved treatment of other persistent and biofilm infections in general.

## 1. Introduction

As a virulent opportunistic pathogen, *Staphylococcus aureus* is the most common cause of skin infections and can also cause chronic persistent infections such as endocarditis and osteomyelitis [1,2,3]. For conditions, such as endocarditis and osteomyelitis, and prosthetic joint infections, treatments with vancomycin as a monotherapy or drug combination for at least six weeks are recommended. Drug combinations, such as doxycycline + rifampin for up to 10 days, vancomycin + gentamicin + rifampin for at least six weeks, are recommended to treat chronic infections, such as recurrent tissue infections and endocarditis on prosthetic valves, respectively (The Johns Hopkins Antibiotics Guide). In particular, indwelling devices are conducive to biofilm formation, complicating treatment and leading to prolonged infections. Up to 80% of human infections are biofilm infections, and globally, chronic persistent and biofilm infections represent a huge burden to public health as they increase the length of hospital stay, cause relapse, cost of treatment, and risk of death by at least three-fold [4]. Bacteria in biofilms are more tolerant to antibiotics compared to planktonic cells [5]. Studies have shown that antibiotics can penetrate the biofilm but they do not always kill the bacteria, suggesting that tolerance to treatment is not due to impaired antibiotic penetration or genetic resistance [6,7], but due to dormant non-growing or slow growing persister bacteria. Bacteria inside the biofilm are quite heterogeneous as some cells grow slowly, which are representative of stationary phase bacteria, while others form dormant persister cells due to the high cell density, nutrient, and oxygen limiting environment inside the biofilm matrix [8].

Persisters were first described in 1942 by Hobby et al., who found that while 99% of *S. aureus* cells were killed by penicillin, about 1% of residual metabolically quiescent or dormant cells called persisters were not killed [9]. The persisters were not resistant to penicillin, and hence did not undergo genetic changes, but were phenotypic variants that became tolerant to antibiotics [10]. Similarly, a clinical observation was also made as penicillin failed to clear chronic infections due to the presence of persister cells found in patients [10]. While the mechanisms of *S. aureus* persistence were largely unknown for a long time, recent studies have shown that pathways involved in quorum sensing, pigmentation production, and metabolic processes, such as oxidative phosphorylation, glycolysis, amino acid, and energy metabolism [11,12,13,14,15], are involved. Despite the observation of persister bacteria from 1940s and their implications in causing prolonged treatment and post-treatment relapse, the importance of persister bacteria and drugs that target persister bacteria in clinical settings has been ignored largely because no persister drugs have been found that can cure or shorten treatment duration or reduce relapse in clinically relevant persistent infections. The importance of persister drugs to more effectively cure persistent infections is only recognized in the case of tuberculosis persister drug pyrazinamide (PZA), which shortens the treatment from 9–12 months to six months after its inclusion in a drug combination setting [16]. PZA’s activity in killing *M. tuberculosis* persisters, unlike the other drugs used to treat tuberculosis, is crucial in developing a shorter treatment due to its unique mechanisms of action by inhibiting persister targets, including energy metabolism (CoA synthesis via PanD) and protein degradation pathways (RpsA and ClpC1) essential for persister survival [16,17,18,19]. The drug PZA validates an important principle of using a persister drug in combination with other drugs targeting both non-growing persisters and growing bacteria in formulating an effective therapy for chronic persistent infections [20,21]. In support of this idea, more recently, a similar approach was used to identify the effective drug combination using persister drug daptomycin in combination with doxycycline and cefuroxime, which completely eradicated biofilm-like structures of *Borrelia burgdorferi* in vitro [22] and in mice [23].

Using this approach, in a recent study aimed at identifying drugs targeting non-growing persisters, we used a stationary phase culture of *S. aureus* as a drug screen model and identified several drugs, such as clinafloxacin and tosufloxacin, with high activity against *S. aureus* persisters [24]. However, their activities alone and in drug combinations in killing biofilms have not been evaluated in vitro or in related *S. aureus* persistent infections in vivo. In this study, we developed persister drug combinations utilizing persister drug clinafloxacin that can more effectively eradicate *S. aureus* biofilms by formulating drug combinations that have high activities against growing bacteria and non-growing persisters in a biofilm model in vitro initially. Then, we established a persistent skin infection mouse model for *S. aureus* using biofilm “persister seeding” [21,25] and evaluated drug combinations in clearing the biofilm infection in this persistent infection model. Here, we show that combining meropenem and daptomycin targeting growing bacteria, with persister drug clinafloxacin targeting biofilm persister bacteria led to the complete eradication of *S. aureus* biofilms not only in vitro, but more importantly also in vivo in a murine model of persistent skin infection, whereas other approaches for treating persistent infections and the currently recommended drug combination treatment without persister drugs failed to do so.

## 2. Materials and Methods

### 2.1. Culture Media, Antibiotics, and Chemicals

*Staphylococcus aureus* strains Newman, USA300 (a biofilm-proficient common circulating strain of community acquired-MRSA, CA-MRSA) and clinical strains CA-409, CA-127, and GA-656 were obtained from American Type Tissue Collections (Manassas, VA, USA) and cultivated in tryptic soy broth (TSB) and tryptic soy agar (TSA) (Becton Dickinson, Franklin Lakes, NJ, USA) at 37 °C. Vancomycin, gentamicin, rifampicin, levofloxacin, ciprofloxacin, moxifloxacin, and oritavancin were obtained from Sigma-Aldrich Co. (St. Louis, MO, USA). Daptomycin, meropenem, tosufloxacin, and clinafloxacin were obtained from AK Scientific, Inc. (Union City, CA, USA). Stock solutions were prepared in the laboratory, filter-sterilized, and used at indicated concentrations. 

### 2.2. Microtiter Plate Biofilm Drug Exposure Assay and the SYBR Green I/PI Assay

*S. aureus* strains grown overnight in TSB were diluted 1:100 and 100 μL aliquots of each diluted culture, placed into a 96-well flat-bottom microtiter plate, and statically incubated for 24 h at 37 °C [26]. Planktonic cells were removed and discarded from the microtiter plates. Drugs at the indicated Cmax concentrations were then added to the biofilms attached to the bottom of the microtiter plate in a total volume of 100 μL in TSB medium and incubated at 37 °C without shaking for 4 days. To determine the cell and biofilm density, the supernatant was removed from the well and the biofilms were washed twice with PBS (1×). To enumerate bacterial cell counts, the biofilms in the wells were resuspended in TSB and scraped with a pipette tip before serial dilution and plating on TSA plates for CFU counts. 

SYBR Green I/PI assay was used to assess biofilm cell viability using the ratio of green:red fluorescence to determine the ratio of live:dead cells, respectively, as described [27]. Briefly, the staining dyes were prepared by mixing SYBR Green I/PI (10,000× stock, Invitrogen, Carlsbad, CA, USA) with propidium iodide (20 mM, Sigma-Aldrich) in distilled water at a ratio 1:3 in 100 µL distilled H_2_O. The SYBR Green I/PI dye mix (10 µL) was added to each 100 µL of sample and incubated at room temperature in the dark for 20 min. With excitation wavelengths of 485 nm and 538 nm and 612 nm for green and red emission, respectively, the green and red fluorescence intensity was determined for each sample using a Synergy H1 microplate reader by BioTek Instruments (Winooski, VT, USA).

### 2.3. Mouse Skin Infection Model

Female Swiss-Webster mice of 6 weeks of age were obtained from Charles River. They were housed 3–5 per cage under BSL-2 housing conditions. All animal procedures were approved by the Johns Hopkins University Animal Care and Use Committee (protocol code MO17H167 and 23 May 2017 approval). *S. aureus* strain USA300 and strain Newman were used in the mouse infection experiments. The details of the biofilm mouse infection model were described in our previous study [25]. Briefly, mice were anesthetized and then shaved to remove a patch of fur of approximately 3 cm by 2 cm. For the preparation of biofilm inoculum for infection, biofilms were first grown in microtiter plates as we described previously [25], and then resuspended and scraped up with a pipette tip. Quantification of all inoculum was performed by serial dilution and plating. Bacteria of indicated inoculum size (10^8^ CFU/mL) were subcutaneously injected into the mice. Treatment was started after 1 week of infection with different drugs and drug combinations for 1 week. For details on drugs, drug dosage, and route of administration, please refer to Table 1. Skin lesion sizes were measured at the indicated time points using a caliper. The area of the lesion size was calculated by measuring the lesion as an oval shape [area = π × (length/2) × (width/2)]. Mice were euthanized after 1 week post-treatment, and skin tissues were removed, homogenized, and serially diluted for bacterial counting on TSA plates.

### 2.4. Histopathology

Skin tissues were dissected, laid flat, and fixed for 24 hrs with neutral buffered formalin. Tissues were embedded in paraffin, cut into 5-μm sections, and mounted on glass slides. Tissue sections were stained with hematoxylin and eosin for histopathological scoring. Tissue sections were evaluated for lesion crust formation, ulcer formation, hyperplasia, inflammation, gross size, and bacterial count and were assigned a score on a 0–3 scale (0 = none, 1 = mild, 2 = moderate, and 3 = severe). The cumulative pathology score represented the sum of each individual pathology parameter. Scoring was performed by an observer in consultation with a veterinary pathologist. Representative images were taken using a Keyence BZ-X710 Microscope (Itasca, IL, USA). 

### 2.5. Statistical Analyses

Statistical analyses were performed using ANOVA or Student’s *t*-test where appropriate. Mean differences were considered statistically significant if the *p* value was <0.05. All experiments were performed in triplicates. Analyses were performed using GraphPad Prism version 8.0.2 and Microsoft Office Excel 2016.

## 3. Results

### 3.1. Commonly Used Treatments for MRSA Have Poor Activity against Biofilms In Vitro 

**Figure 1:**
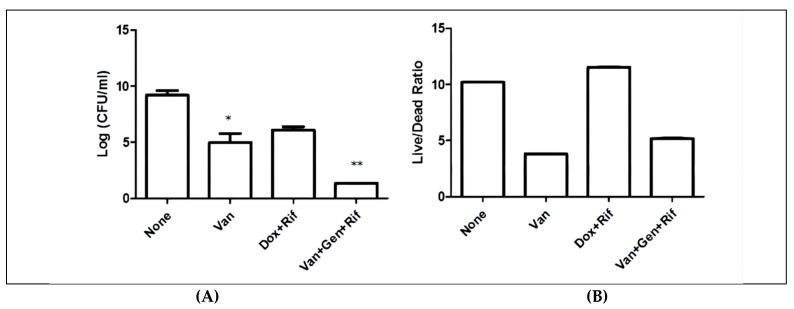
Clinically recommended treatments for chronic *S. aureus* infections only partially killed *S. aureus* USA300 biofilm bacteria in vitro. Treatments (all 50 μM) of vancomycin alone, doxycycline + rifampin, and the combination of vancomycin + gentamicin + rifampin for 4 days were evaluated for biofilm killing by CFU enumeration (**A**) and viability staining using SYBR Green I/PI (**B**). See Methods section for details. Vancomycin, Van; Doxycycline, Dox; Rifampin, Rif; Gentamicin, Gen. Student’s *t*-test, * *p*  <  0.05, ** *p*  <  0.005. Error bars indicate standard deviation.

We first evaluated the activity of the above drugs in killing biofilm bacteria in vitro using traditional bacterial CFU counts (Figure 1A) and viability assessment by SYBR Green I/PI staining that has been developed to screen for drugs targeting Borrelia persister bacteria [27] (Figure 1B). We found that such clinically used combinations were not completely effective against biofilms, as shown by different assays, i.e., CFU assay (Figure 1A), SYBR Green/PI assay (Figure 1B). After four days of treatment, biofilm bacteria were not completely eradicated by any of the current treatments with vancomycin alone, or doxycycline + rifampin or vancomycin + gentamicin + rifampin as shown by significant numbers of bacteria remaining (Figure 1). However, of note, vancomycin + gentamicin + rifampin was more active than vancomycin alone or doxycycline + rifampin in killing biofilm bacteria (Figure 1A).

### 3.2. Identification of Drug Combinations with Strong Anti-Biofilm Activity

**Figure 2:**
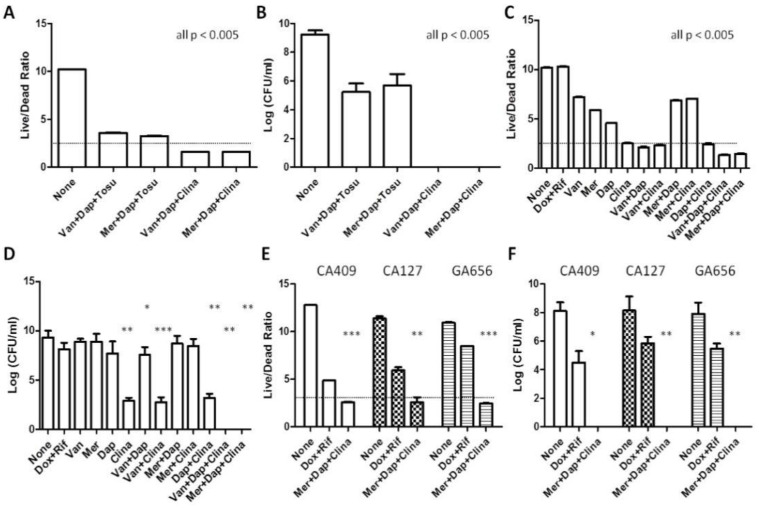
Identification of drug combinations that kill MRSA biofilms. Various drug combinations consisting of drugs (at 50 μM) highly active against the growing phase and persister cells were incubated with *S. aureus* USA300 biofilm bacteria for 4 days followed by assessing the anti-biofilm activity using SYBR Green I/PI viability staining (**A**) and CFU enumeration (**B**). Drug combinations with sterilizing activity against USA300 biofilms were tested at clinically achievable Cmax concentrations using SYBR Green I/PI viability staining (**C**) and CFU enumeration (**D**). Validation of meropenem + daptomycin + clinafloxacin in killing biofilms of various MRSA clinical isolates (**E**,**F**). Vancomycin, Van; Meropenem, Mer; Daptomycin, Dap; Tosufloxacin, Tosu; Clinafloxacin, Clina; Doxycycline, Dox; Rifampin, Rif. One-way ANOVA and post hoc Tukey’s test was used for multiple group comparisons. * *p*  <  0.05, ** *p * <  0.005, *** *p * <  0.0005. Error bars indicate standard deviation.

To address the clinical unmet need for better treatments for persistent biofilm infections, we hypothesize that a drug combination that includes drugs that act on growing bacteria, such as cell wall (e.g., vancomycin, meropenem) or cell membrane inhibitors (e.g., daptomycin), plus a drug that acts on persister bacteria will be more potent in eradicating biofilm bacteria. Previous studies from our lab identified tosufloxacin and clinafloxacin as having strong anti-persister activity against *S. aureus* [24]. In order to identify a potent combination, we tested various drug combinations that include drugs against both growing bacteria and non-growing persisters in a biofilm model with *S. aureus* USA300, a common biofilm-proficient clinical MRSA strain. While we previously showed that tosufloxacin had robust activity against *S. aureus* persister cells [24], the drug combination of vancomycin/meropenem + daptomycin + tosufloxacin achieved only partial eradication, with 10^5^ CFU/mL biofilm bacteria remaining after treatment (Figure 2A,B). In contrast, the combination of vancomycin/meropenem + daptomycin + clinafloxacin showed complete eradication of biofilms after 4-day treatment, as shown by 0 CFU and a live/dead ratio below the limit of detection in SYBR Green/PI assay (Figure 2A,B). Although we used the same molar concentration of each individual drug at 50 μM for comparison of relative drug activity, to evaluate the activity of the drug combination in a more clinically relevant manner, we treated the biofilms with the drugs at their Cmax concentrations (Table 1). Since the drugs used in the anti-biofilm studies are mostly clinically used drugs, they would not be expected to have significant cytotoxicity at their respective Cmax (maximum human blood) drug concentrations. Our findings with Cmax drug concentrations were confirmatory as the drug combination of vancomycin/meropenem + daptomycin + clinafloxacin still achieved complete eradication, while drug-free control and the clinically used combination of doxycycline + rifampin could not. The clearance of biofilms was confirmed by both CFU counts and the SYBR Green I/PI viability stain (Figure 2C,D).

We then tested the potential of the drug combination of meropenem + daptomycin + clinafloxacin to eradicate biofilm bacteria from different MRSA *S. aureus* strains, including CA-MRSA clinical isolates CA-409, CA-127, and hospital-acquired MRSA strain GA-656. Complete eradication (0 CFU/mL) and undetectable levels of live cells (under the limit of detection) were found for all of the MRSA strains tested after 4 days of treatment with the combination meropenem + daptomycin + clinafloxacin (Figure 2E,F).

### 3.3. Unique Anti-Persister Activity of Clinafloxacin Could Not Be Replaced by Other Fluoroquinolone Drugs

**Figure 3:**
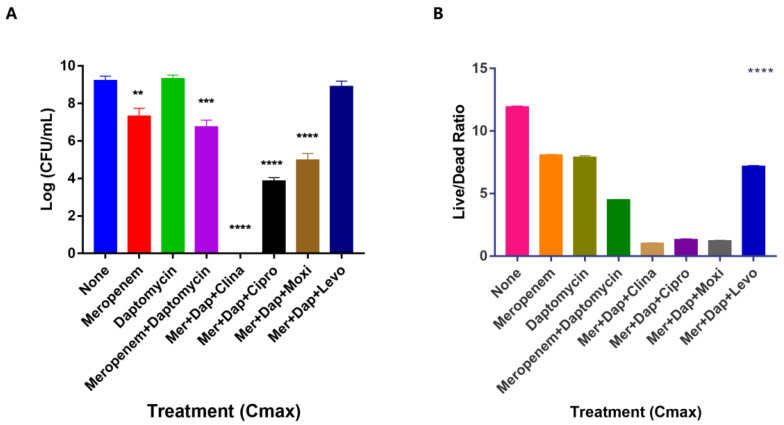
Ranking of fluoroquinolones in drug combinations at Cmax concentrations for activity against *S. aureus* USA300 biofilm bacteria. Commonly used fluoroquinolones ciprofloxacin, moxifloxacin, levofloxacin were evaluated together with clinafloxacin in combination with meropenem+daptomycin in drug exposure tests for 4 days with *S. aureus* Newman biofilm bacteria, when the effect of the treatment was assessed by (**A**) CFU count and (**B**) SYBR Green/PI viability assay as described in Methods. Meropenem, Mer; Daptomycin, Dap; Clinafloxacin, Clina; Ciprofloxacin, Cipro; Moxifloxacin, Moxi; Levofloxacin, Levo. One-way ANOVA and post hoc Tukey’s test was used for multiple group comparisons. ** *p*  <  0.05, *** *p * <  0.005, **** *p * <  0.0001. Error bars indicate standard deviation.

Clinafloxacin is a member of the fluoroquinolone antibiotics which inhibits DNA replication by binding to DNA gyrase. As our results suggest (Figure 2D), clinafloxacin is a powerful anti-persister drug. Hence, we wanted to rank the anti-biofilm activity of different fluoroquinolones to determine whether the robust anti-biofilm activity of clinafloxacin is unique or can be replaced by other fluoroquinolones by using CFU assay and SYBR Green/PI viability assay. We used the *S. aureus* Newman strain due to its susceptibility to many fluoroquinolones to avoid any confounding factors due to inherent drug resistance. While other fluoroquinolones such as ciprofloxacin, levofloxacin, and moxifloxacin had certain anti-biofilm activity when used in combination with meropenem and daptomycin after four days of treatment, the drug combination with clinafloxacin was indeed the most active and was the only combination that achieved complete sterilization as seen by CFU count (Figure 3A). By contrast, biofilms treated with other quinolone (ciprofloxacin, moxifloxacin, levofloxacin) combinations still had 10^4^–10^8^ CFU/mL bacteria remaining (Figure 3A). The results with the SYBR Green/PI viability assay, where lower live/dead ratios would indicate lower number of viabile bacteria, were consistent with the CFU assay. However, the SYBR Green/PI assay, while being quick and showing general agreement with the CFU assay, was not as discriminative or accurate as the CFU assay (Figure 3B) as it could not distinguish between Mer+Dap+Clina, Mer+Dap+Cipro, and Mer+Dap+Moxi as the assay reached the limit of detection near live/dead ratios of 1–2 (Figure 3B). When used in combination, the activity of the quinolones from strongest to weakest as ranked by both viability assessment and viable cell counts is as follows: clinafloxacin, ciprofloxacin, moxifloxacin, and levofloxacin (Figure 3). Hence, clinafloxacin has unique potent activity against biofilm bacteria compared to other fluoroquinolone counterparts.

### 3.4. Anti-Biofilm Activity of Oritavancin and Dalbavancin

**Figure 4:**
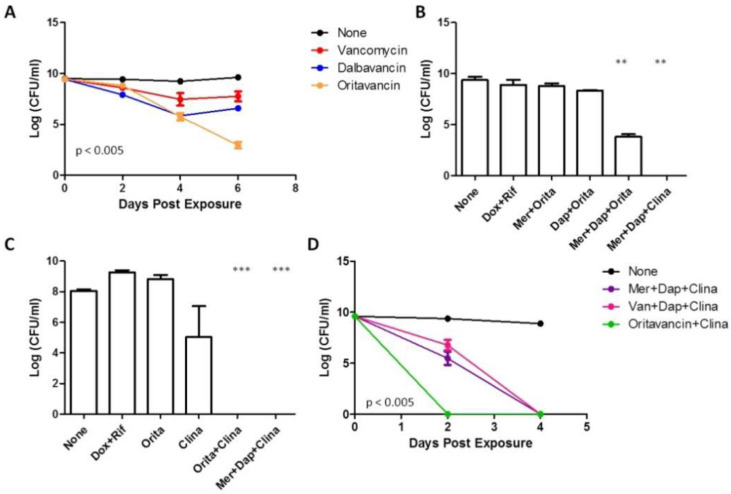
Evaluation of oritavancin in killing *S. aureus* USA300 biofilms as a single drug or in combinations. (**A**) Comparison of novel lipoglycopeptides oritavancin and dalbavancin with vancomycin (at 50 μM) in their activity to kill biofilm bacteria. (**B**) Evaluating oritavancin in killing persisters in combination with meropenem + daptomycin, and (**C**) in killing growing phase bacteria in combination with clinafloxacin (at Cmax concentrations). (**D**) Time-kill curve of biofilms comparing the top drug combination candidates (at Cmax concentrations). Meropenem, Mer; Daptomycin, Dap; Oritavancin, Orita; Clinafloxacin, Clina; Doxycycline, Dox; Rifampin, Rif. One- or two-way ANOVA and post hoc Tukey’s test was used for multiple group comparisons., ** *p*  <  0.005, *** *p * <  0.0005. Error bars indicate standard deviation.

Thus far, our data suggest that the inclusion of a drug with great anti-biofilm activity can be beneficial in killing biofilm bacteria (Figure 2 and Figure 3). To identify other potential anti-biofilm drug candidates, we turned to the new generation of lipoglycopeptides such as oritavancin and dalbavancin. These drugs have multiple mechanisms of action: inhibition of transglycosylation, transpeptidation, and cell membrane disruption, a property of persister drugs [17]. We first tested the activity of oritavancin and dalbavancin in killing *S. aureus* biofilm bacteria in comparison with their parent compound vancomycin, and the results revealed oritavancin was the best in killing biofilm among the three drugs (Figure 4A). After six days of drug exposure, oritavancin killed 10^6^ CFU/mL bacteria as compared with dalbavancin or vancomycin, which killed only about 10^2^ CFU/mL.

Since oritavancin showed strong anti-biofilm activity, we next evaluated oritavancin’s activity in drug combinations. After replacing clinafloxacin with oritavancin, we observed that the combination of meropenem + daptomycin + oritavancin exhibited partial activity against biofilms, causing a decrease of 10^5^ CFU/mL, which is much better than the activity achieved by single drugs or two-drug combinations, but still was inferior to the clinafloxacin combination (Figure 4B).

Due to oritavancin’s strong activity against growing *S. aureus* (MIC = 0.03 mg/L) [28,29] and its dual mechanism of action that mimics cell wall + cell membrane inhibitor, we tested oritavancin in place of meropenem and daptomycin. Surprisingly, the combination of oritavancin + clinafloxacin was also able to achieve complete eradication of biofilms suggesting that oritavancin can replace the drugs against growing bacteria (Figure 4C). It is also important to note that oritavancin alone could not kill biofilms (no change in CFU after 4-day treatment), which further validates the importance of drug combinations for biofilm bacteria.

To compare the activity of the three combinations tested thus far with clinafloxacin, we performed a time-kill experiment which revealed that oritavancin + clinafloxacin could kill all biofilms by day 2 of treatment whereas it took four days for meropenem/vancomycin + daptomycin + clinafloxacin to eradicate the biofilm bacteria (Figure 4D). Overall, our data suggest that the inclusion of an anti-biofilm drug in a drug combination to treat biofilms is paramount and these combinations possess much better activity than current clinically used regimens based on our in vitro studies (Figure 4B,C). 

### 3.5. The Drug Combination Meropenem + Daptomycin +Clinafloxacin Eradicated Biofilm Infections in the Mouse Skin Persistent Infection Model

**Figure 5:**
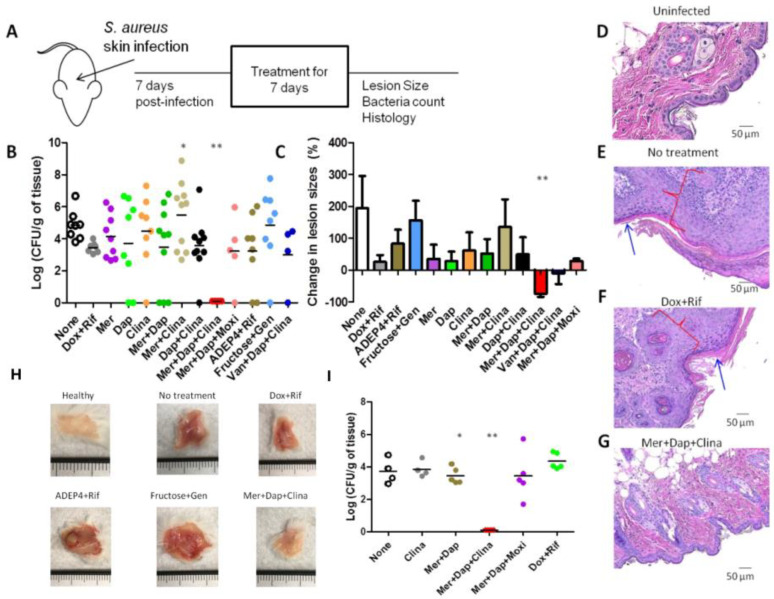
Validation of drug combinations in a chronic skin infection model established with *S. aureus* USA300 biofilm bacteria. (**A**) Study design of mouse treatment studies. (**B**) Bacterial load in the skin lesions. (**C**) Changes in lesion sizes and histology of skin tissues of mice infected with USA300 biofilm bacteria and treated 7-days with drug combinations and respective controls were measured. Histopathology of uninfected mice (**D**), infected mice receiving 7-days of no treatment (**E**), or treated with doxycycline + rifampin (**F**), or treated with meropenem + daptomycin + clinafloxacin (**G**) was analyzed. (**H**) Gross pathology lesions of skin tissues of mice treated 7-days with drug combinations and respective controls (in mm). (**I**) Bacterial loads in the skin tissues of mice infected with MSSA Newman strain and treated for 7-days with drug combinations or control treatments were enumerated. Images were taken at 200 X magnification. Meropenem, Mer; Daptomycin, Dap; Clinafloxacin, Clina; Doxycycline, Dox; Rifampin, Rif, Gentamicin, Gen. Blue arrows indicate crust formation, red brackets indicate hyperplasia and cellular infiltration. One-way ANOVA and post hoc Tukey’s test was used for multiple group comparisons. * *p*  <  0.05, ** *p*  <  0.005. Error bars indicate standard deviation.

Given the robust activity of our drug combinations in eradicating biofilms in vitro, we were interested to know if the drug combination could also eradicate persistent infections in vivo. To do so, we chose to infect mice with biofilm bacteria from *S. aureus* strain USA300, a clinical MRSA strain most representative to cause persistent infections in humans. We allowed the infection to develop for seven days, followed by seven days of treatment with different regimens (Figure 5A). Previously, we have shown that mice infected with biofilm bacteria developed more chronic skin lesions [25]. Administration of the combination of doxycycline + rifampin (a control group as a clinically used treatment) or drug combination vancomycin + daptomycin + clinafloxacin decreased the bacteria load (about 1-log of bacteria) but did not clear the infection (Figure 5B). Other reported treatments which supposedly eradicate chronic *S. aureus* infections, such as ADEP4 + rifampin [30] or gentamicin + fructose [31], did not show sterilizing activity in our biofilm infection model, but instead caused increased lesion size and inflammation (Figure 5C,H). Remarkably, the combination of meropenem + daptomycin + clinafloxacin cleared the infection completely, decreased the size of lesions, reduced histopathology scores, and healed the lesions completely (Figure 5B,C,G), while other treatments, such as the control treatment with doxycycline + rifampin still had considerable lesions (Figure 5B,C,F). Intriguingly, in gross lesion examination, consistent with the lesion size data in Figure 5C, ADEP4 + rifampin [30] or gentamicin + fructose [31] produced worsened lesions (Figure 5H). 

To verify the above observation, we performed a separate study with a methicillin-susceptible *S. aureus* (MSSA) Newman strain and compared clinafloxacin’s activity with other quinolones. We were able to confirm the above findings with the MRSA strain and found that despite moxifloxacin and clinafloxacin having the same MIC for the Newman strain, the combination of meropenem + daptomycin + moxifloxacin was not effective in clearing the biofilm infection. In contrast, the meropenem + daptomycin + clinafloxacin combination indeed eradicated the infection completely (Figure 5I). This indicates clinafloxacin combination works for both MRSA and MSSA strains, and the unique sterilizing activity of clinafloxacin cannot be replaced by moxifloxacin. The above data support our hypothesis that a drug combination targeting both growing (e.g., meropenem, daptomycin) and persister bacteria (e.g., clinafloxacin) is essential in clearing chronic infections in vivo, such as the persistent skin infection caused by *S. aureus* biofilm.

## 4. Discussion

Numerous studies have documented how resilient biofilms are to antibiotic treatments [32,33,34,35]. Since persister cells that are embedded in the biofilm are mostly responsible for the recalcitrance of biofilms to antibiotic treatments, many attempts have been made to identify novel effective treatments and synthetic compounds that kill bacterial persisters [36,37]. Some approaches include resuscitating or altering the metabolic status of persisters [38,39] or enhancing the activity of aminoglycoside antibiotics with sugars such as fructose [31] or activating protease by ADEP4 plus rifampin [30]. Although these new therapeutic approaches showed promising results in vitro and in some cases in animal models [30,36,40], not all treatments achieved complete sterilization and their utility in more persistent biofilm infections remains to be demonstrated. The animal models used either rely on immunosuppressant agents or short-term infections [30,31,36], which do not reflect true persistent infections clinically. Here, we used a recently developed more relevant persistent infection mouse model using biofilm inocula that mimic human infections without the use of immunosuppressant agents [25] for evaluation of drug combinations. Previous studies have mostly used log phase bacteria as inocula for infection in animal models [25]. However, we showed that *S. aureus* biofilm inocula produced a more severe lesion and more persistent infection than the log phase bacteria and the current treatments failed to eradicate the biofilm infection [25]. This biofilm-inocula model could serve as a useful model for evaluating treatment regimens against biofilm infections in vivo in general. Importantly, we demonstrate that the persister drug combination meropenem + daptomycin + clinafloxacin completely eradicated the biofilm infection in the mouse model, while the currently recommended treatment doxycycline+rifampin or daptomycin+meropenem and the well-known experimental persister treatment regimens, such as ADEP4+rifampin [30] or gentamicin+fructose [31], failed to do so (Figure 5B). To our knowledge, this is the first time a biofilm infection is completely eradicated by a specific persister drug combination but not by the current clinically used standard antibiotic treatments or other known experimental treatments, such as ADEP4+rifampin [30] or gentamicin+fructose [31], for persistent infections.

The mechanisms by which combination treatment effectively eradicates biofilm-derived bacteria are worth commenting upon. The well-known experimental regimens for killing persisters and treating persistent biofilm infections include ADEP4+rifampin [30] and gentamicin+fructose [31]. ADEP4 is a ClpP protease activator that kills persisters by degrading numerous cellular proteins, forcing cells to self-digest, and its combination with rifampin was shown to completely eradicate *S. aureus* biofilms in vitro and in a mouse model [30]. The other experimental approach to kill persisters is to use fructose to cause increased generation of proton-motive force (PMF) which facilitates aminoglycoside uptake for enhanced drug activity [31]. However, the highly potent anti-biofilm agent clinafloxacin seems to work differently. We previously hypothesized, per the Yin–Yang model, that a drug combination approach using drugs targeting different bacterial populations, i.e., non-growing persisters (Yin) and growing bacteria (Yang), will be required to more effectively cure persistent and biofilm infections [21]. Thus, it is likely that the sterilizing activity of the clinafloxacin drug combination (meropenem + daptomycin + clinafloxacin) in eradicating the biofilm bacteria in vitro and persistent skin infections in mice is due to the combined action of the unique anti-persister activity of clinafloxacin targeting persisters in combination with meropenem + daptomycin targeting growing bacteria. The strong activity of meropenem (cell wall inhibitor) and daptomycin (cell membrane disruptor) against *S. aureus* growing bacteria [41,42] allows for the rapid killing of growing bacteria in the biofilm bacterial population. While meropenem and daptomycin are directed at killing growing bacteria, they also have certain activity against persisters. Meropenem used in combination with polymyxin B has been shown to eradicate persisters in *Acinetobacter baumannii* [43]. Similarly, daptomycin has activity against *S. aureus* biofilms [44]. Daptomycin in combination with doxycycline and cefoperazone or cefuroxime has been shown to kill biofilm-like microcolony persisters of B. burgdorferi in vitro and in mice [23,45]. Daptomycin could disrupt the bacterial membrane structure and cause rapid depolarization of the membrane [46], which may affect the viability of some persisters. Nevertheless, Daptomycin alone or with other drug combinations had limited activity against *S. aureus* biofilm bacteria and could not achieve sterilization (Figure 2 and Figure 5). To kill non-growing biofilm persisters, clinafloxacin has been shown to be crucial in the combination as moxifloxacin or levofloxacin in place of clinafloxacin in the above combination failed to eradicate the biofilm bacteria in vitro and in mice (Figure 3A, Figure 5B). This occurs despite the MICs for clinafloxacin and moxifloxacin for *S. aureus* being the same, yet they differ in their activity to kill persisters both singly and in drug combinations both in vitro and in mice (Figure 5B,I). As a quinolone, clinafloxacin inhibits bacterial DNA gyrase and topoisomerase and thereby interferes with DNA synthesis. However, not all quinolones have significant anti-persister activity [24] (Figure 3). The unique anti-persister and biofilm activity of clinafloxacin seems to be beyond its common mechanism of action on inhibition of DNA synthesis and may be due to its additional chemical groups that attack critical persister targets. Comparing the chemical structure of clinafloxacin to the other quinolones that have relatively weak anti-persister activity (ciprofloxacin, moxifloxacin, and levofloxacin), a chloride group attached to the benzene ring appears to be unique to clinafloxacin and may be responsible for its high anti-biofilm activity. Further studies are required to explore the mechanism of clinafloxacin’s unique ability to kill persisters, including the possibility that clinafloxacin may bind to the target gyrase with higher affinity or it might have preferential activity against targets crucial for persister survival.

It is important to note that the chronic persistent infection status of the mice inoculated with biofilm bacteria is a key component of our disease model. While Conlon et al. used a 1:100 dilution of stationary phase culture (2 × 10^6^) as inocula to infect their mice and caused a deep-seated infection, the infection was allowed to develop for only 24 hours before treatment which cannot be a persistent infection; and the mice were made neutropenic [30], a condition that may not apply to most patients suffering from chronic *S. aureus* infections. Such differences in the animal models may explain why ADEP4 + rifampin, which was claimed to have sterilizing activity and eradicated persistent infection [30], failed to eradicate the more persistent infection in our model established with biofilm inocula and allowed to develop for one week before treatment. In addition, although the combination of an aminoglycoside + sugars has been proposed as an approach for killing persisters and treating persistent infections and was shown to be effective in an E. coli urinary tract infection mouse model [31], this approach was not effective in our biofilm infection model either. Allison et al. showed that gentamicin + fructose reduced 1.5-fold *S. aureus* biofilms in vitro after four hours of treatment [31] but it was not tested in animals. In our study here, we showed that mice treated for seven days with gentamicin + fructose still harbored 10^5^ CFU/mL in skin tissues (Figure 5B). Surprisingly, gentamicin + fructose and ADEP4 + rifampin caused an increase in lesion size despite the treatment (Figure 5C,H), which is hard to explain. In both the above cases, the discrepancy could be due to differences in the disease models, as ours is a more persistent biofilm skin infection model established with biofilm inocula and would be expected to be more difficult to cure than those in the other studies that did not use biofilm inocula for the infection. Although our persistent biofilm infection was established using USA300 strain, a biofilm-proficient clinical MRSA strain, we fully expect that this model can be reproduced with the more clinically essential clones such as ST93, ST80, ST30. We noted that the *S. aureus* Newman strain had a point mutation (Pro18Leu) in the SaeS of the two-component system SaeRS, causing a defect in biofilm formation [47]. However, our study was performed mostly on the biofilm-proficient clinical MRSA strain USA300 and only used the Newman strain for confirmation as a drug susceptible strain control (see Figure 5I). In both cases with USA300 and Newman, the clinafloxacin drug combination (meropenem+daptomycin+clinafloxacin) completely eradicated the biofilm infection (Figure 5B,I). Therefore, there should be no concern about the validity of the results on the eradication of biofilm bacteria by the clinafloxacin drug combination, because the main findings of the study were taken on the USA300 strain (Figure 1, Figure 2, Figure 3, Figure 4, Figure 5, except Figure 5I) but not on the biofilm defective Newman strain. It is worth noting that our focus is to identify more powerful drug combinations that eradicate biofilm infections without any residual surviving bacteria but not on host immune factors that clear the bacterial infection. The near normal or minimal tissue inflammation and pathology after treatment with the most effective regimen meropenem+daptomycin+clinafloxacin (Figure 5G) is meant to show the effectiveness of the treatment in comparison with the standard treatment with doxycycline+rifampin, which still had significant tissue pathology (Figure 5F). Future studies to examine the role of immune control of the persistent biofilm infection in this model would be of interest. 

Clinafloxacin shows impressive anti-biofilm activity in this study and both oral and intravenous formulations have been developed [48,49]. The drugs used in the antibiofilm studies are mostly clinically used drugs, and as such they would not be expected to have significant cytotoxicity at their respective Cmax (maximum human blood drug) concentrations. In addition, clinafloxacin administration drastically improved the condition of a cystic fibrosis patient who had a chronic Burkholderia cenocepacia infection and was not responding to other antibiotic treatments [50]. A human trial with patients having native or prosthetic valve endocarditis also showed that clinafloxacin was an effective treatment [51]. Nevertheless, clinafloxacin is not FDA-approved due to photosensitivity and hypoglycemia. However, the topical use of clinafloxacin or its drug combinations for chronic skin infections would be of interest and could be explored in the future. Further studies with new analogs of clinafloxacin without significant side effects but maintaining high anti-persister activity are needed in the future. Our in vitro data also suggested that oritavancin used in combination with clinafloxacin had robust activity against biofilms, killing all the bacteria after a short treatment of two days. The administration of oritavancin is a single 1200-mg dose given in a slow, three-hour infusion, which may also be of interest for patients due to the ease of administration and long half-life. Hence, further preclinical studies in mice to test oritavancin’s activity in chronic infection models are warranted.

Currently used regimens for treating persistent infections are lengthy and ineffective and the inability to clear the bacteria in a timely fashion may also increase the chance of developing antibiotic resistance. A drug combination approach that targets both growing bacteria and non-growing persister cells as proposed in the Yin–Yang model [21] has promising potential in developing a more effective therapy for treating chronic persistent infections including biofilm infections. This study provides further validation of the Yin–Yang model [21] for treating persistent infections [23,52] and emphasizes the importance of persister drugs such as clinafloxacin in eradicating a persistent infection. This treatment algorithm takes into account the heterogeneous population of bacterial cells that exists upon encountering stress. With this principle in mind, this study reports novel drug combinations that are effective in killing *S. aureus* biofilms and treating chronic infections. In further support of this Yin–Yang model of targeting both growing and non-growing bacteria for more effective treatment of persistent infections, we also demonstrated, in a separate study, the complete eradication of a different persistent pulmonary infection with Pseudomonas aeruginosa in a mouse model of cystic fibrosis [52].

In conclusion, we showed that commonly used treatments for *S. aureus* infections have poor activity against biofilms in vitro and in vivo, and importantly, we were able to identify promising persister drug clinafloxacin drug combinations, meropenem (or vancomycin) + daptomycin + clinafloxacin, or oritavancin + clinafloxacin, that achieved complete eradication of *S. aureus* biofilm bacteria in vitro. More importantly, we demonstrated that the persister drug combination meropenem + daptomycin + clinafloxacin completely eradicated the bacterial load and healed lesions promptly with reduced pathology and inflammation in the mouse model of persistent biofilm infection, while the current treatments failed to do so. Our approach of combining drugs targeting both growing and non-growing bacteria with persister drugs to completely eradicate biofilm infections may have implications for developing more effective treatments against other persistent infections caused by different bacterial pathogens, fungi, and even cancer.

## Figures and Tables

**Table 1:** Drug dosage, scheduling, and administration.

Drug	Dosage	Route	Times Treated	Cmax Tested (Clinically Achievable Concentrations) *
Vancomycin	110 mg/kg	Intraperitoneally	Twice/daily	20 µg/mL
Daptomycin	50 mg/kg	Intraperitoneally	Once/daily	80 µg/mL
Meropenem	50 mg/kg	Intraperitoneally	Once/daily	20 µg/mL
Clinafloxacin	50 mg/kg	Intraperitoneally	Once/daily	2 µg/mL
Doxycycline	100 mg/kg	Oral	Twice/daily	5 µg/mL
Rifampin	10 mg/kg	Oral	Twice/daily	5 µg/mL
Moxifloxacin	100 mg/kg	Oral	Once/daily	4 µg/mL
ADEP4	25 mg/kg and 35 mg/kg	Intraperitoneally	Twice/daily	
Rifampin (for ADEP4 combination)	30 mg/kg	Intraperitoneally	Once/daily	
Gentamicin	20 mg/kg	Intraperitoneally	Once/daily	
Fructose	1.5 g/kg	Intraperitoneally	Once/daily	
Oritavancin	---	---	---	5 µg/mL
Ciprofloxacin	---	---	---	10 µg/mL
Levofloxacin	---	---	---	10 µg/mL

* Cmax concentrations refer to maximum blood drug concentrations in humans and were derived from Johns Hopkins Antibiotics Guides (https://www.hopkinsguides.com/hopkins/index/Johns_Hopkins_ABX_Guide/Antibiotics, accessed on 20 June 2019).

## Data Availability

Not applicable.

## References

[1] Chan L.C., Chaili S., Filler S.G., Miller L.S., Solis N.V., Wang H., Johnson C.W., Lee H.K., Diaz L.F., Yeaman M.R. (2017). Innate Immune Memory Contributes to Host Defense against Recurrent Skin and Skin Structure Infections Caused by Methicillin-Resistant *Staphylococcus aureus*. Infect. Immun..

[2] David M.Z., Daum R.S. (2010). Community-associated methicillin-resistant *Staphylococcus aureus*: Epidemiology and clinical consequences of an emerging epidemic. Clin. Microbiol. Rev..

[3] Dryden M.S. (2010). Complicated skin and soft tissue infection. J. Antimicrob. Chemother..

[4] Romling U., Balsalobre C. (2012). Biofilm infections, their resilience to therapy and innovative treatment strategies. J. Intern. Med..

[5] de Oliveira A., Cataneli P.V., Pinheiro L., Moraes Riboli D.F., Benini Martins K., de Souza da Cunha M.L.R. (2016). Antimicrobial Resistance Profile of Planktonic and Biofilm Cells of *Staphylococcus aureus* and Coagulase-Negative Staphylococci. Int. J. Mol. Sci..

[6] Stewart P.S., Davison W.M., Steenbergen J.N. (2009). Daptomycin rapidly penetrates a Staphylococcus epidermidis biofilm. Antimicrob. Agents Chemother..

[7] Kirker K.R., Fisher S.T., James G.A. (2015). Potency and penetration of telavancin in staphylococcal biofilms. Int. J. Antimicrob. Agents.

[8] Kavanaugh J.S., Horswill A.R. (2016). Impact of Environmental Cues on Staphylococcal Quorum Sensing and Biofilm Development. J. Biol. Chem..

[9] Hobby G.L., Meyer K., Chaffee E. (1942). Observations on the mechanism of action of penicillin. Proc. Soc. Exp. Biol. Med..

[10] Bigger J.W. (1944). Treatment of staphylococcal infections with penicillin by intermittent sterilisation. Lancet.

[11] Wang W., Chen J., Chen G., Du X., Cui P., Wu J., Zhao J., Wu N., Zhang W., Li M. (2015). Transposon Mutagenesis Identifies Novel Genes Associated with *Staphylococcus aureus* Persister Formation. Front. Microbiol..

[12] Conlon B.P., Rowe S.E., Gandt A.B., Nuxoll A.S., Donegan N.P., Zalis E.A., Clair G., Adkins J.N., Cheung A.L., Lewis K. (2016). Persister formation in *Staphylococcus aureus* is associated with ATP depletion. Nat. Microbiol..

[13] Yee R., Cui P., Shi W., Feng J., Zhang Y. (2015). Genetic Screen Reveals the Role of Purine Metabolism in *Staphylococcus aureus* Persistence to Rifampicin. Antibiotics.

[14] Xu T., Wang X.Y., Cui P., Zhang Y.M., Zhang W.H., Zhang Y. (2017). The Agr Quorum Sensing System Represses Persister Formation through Regulation of Phenol Soluble Modulins in *Staphylococcus aureus*. Front. Microbiol..

[15] Sahukhal G.S., Pandey S., Elasri M.O. (2017). msaABCR operon is involved in persister cell formation in *Staphylococcus aureus*. BMC Microbiol..

[16] Zhang Y., Mitchison D. (2003). The curious characteristics of pyrazinamide: A review. Int. J. Tuberc. Lung Dis..

[17] Zhang Y., Wade M.M., Scorpio A., Zhang H., Sun Z. (2003). Mode of action of pyrazinamide: Disruption of *Mycobacterium tuberculosis* membrane transport and energetics by pyrazinoic acid. J. Antimicrob. Chemother..

[18] Shi W., Zhang X., Jiang X., Yuan H., Lee J.S., Barry C.E., Wang H., Zhang W., Zhang Y. (2011). Pyrazinamide inhibits trans-translation in *Mycobacterium tuberculosis*. Science.

[19] Zhang S., Chen J., Shi W., Liu W., Zhang W., Zhang Y. (2013). Mutations in panD encoding aspartate decarboxylase are associated with pyrazinamide resistance in *Mycobacterium tuberculosis*. Emerg. Microbes Infect..

[20] Zhang Y., Shi W., Zhang W., Mitchison D. (2013). Mechanisms of Pyrazinamide Action and Resistance. Microbiol. Spectr..

[21] Zhang Y. (2014). Persisters, Persistent Infections and the Yin-Yang Model. Emerg. Microbes Infect..

[22] Feng J., Weitner M., Shi W., Zhang S., Zhang Y. (2016). Eradication of Biofilm-Like Microcolony Structures of Borrelia burgdorferi by Daunomycin and Daptomycin but not Mitomycin C in Combination with Doxycycline and Cefuroxime. Front. Microbiol..

[23] Feng J., Li T., Yee R., Yuan Y., Bai C., Cai M., Shi W., Embers M., Brayton C., Saeki H. (2019). Stationary phase persister/biofilm microcolony of Borrelia burgdorferi causes more severe disease in a mouse model of Lyme arthritis: Implications for understanding persistence, Post-treatment Lyme Disease Syndrome (PTLDS), and treatment failure. Discov. Med..

[24] Niu H., Cui P., Yee R., Shi W., Zhang S., Feng J., Sullivan D., Zhang W., Zhu B., Zhang Y. (2015). A Clinical Drug Library Screen Identifies Tosufloxacin as Being Highly Active against *Staphylococcus aureus* Persisters. Antibiotics.

[25] Yee R., Yuan Y., Shi W., Brayton C., Tarff A., Feng J., Wang J., Behrens A., Zhang Y. (2019). Infection with persister forms of *Staphylococcus aureus* causes a persistent skin infection with more severe lesions in mice: Failure to clear the infection by the current standard of care treatment. Discov. Med..

[26] O’Toole G.A. (2011). Microtiter dish biofilm formation assay. J. Vis. Exp..

[27] Feng J., Wang T., Zhang S., Shi W., Zhang Y. (2014). An optimized SYBR Green I/PI assay for rapid viability assessment and antibiotic susceptibility testing for Borrelia burgdorferi. PLoS ONE.

[28] Pfaller M.A., Sader H.S., Flamm R.K., Castanheira M., Mendes R.E. (2018). Oritavancin in vitro activity against gram-positive organisms from European and United States medical centers: Results from the SENTRY Antimicrobial Surveillance Program for 2010-2014. Diagn. Microbiol. Infect. Dis..

[29] Yan Q., Karau M.J., Patel R. (2018). In vitro activity of oritavancin against biofilms of staphylococci isolated from prosthetic joint infection. Diagn. Microbiol. Infect. Dis..

[30] Conlon B.P., Nakayasu E.S., Fleck L.E., LaFleur M.D., Isabella V.M., Coleman K., Leonard S.N., Smith R.D., Adkins J.N., Lewis K. (2013). Activated ClpP kills persisters and eradicates a chronic biofilm infection. Nature.

[31] Allison K.R., Brynildsen M.P., Collins J.J. (2011). Metabolite-enabled eradication of bacterial persisters by aminoglycosides. Nature.

[32] Pinto H., Simões M., Borges A. (2021). Prevalence and Impact of Biofilms on Bloodstream and Urinary Tract Infections: A Systematic Review and Meta-Analysis. Antibiotics.

[33] Høiby N., Bjarnsholt T., Moser C., Bassi G.L., Coenye T., Donelli G., Hall-Stoodley L., Holá V., Imbert C., Kirketerp-Møller K. (2015). ESCMID Study Group for Biofilms and Consulting External Expert Werner Zimmerli. ESCMID guideline for the diagnosis and treatment of biofilm infections 2014. Clin. Microbiol. Infect..

[34] Hughes G., Webber M.A. (2017). Novel approaches to the treatment of bacterial biofilm infections. Br. J. Pharm..

[35] Jacqueline C., Caillon J. (2014). Impact of bacterial biofilm on the treatment of prosthetic joint infections. J. Antimicrob Chemother..

[36] Kim W., Zhu W., Hendricks G.L., Van Tyne D., Steele A.D., Keohane C.E., Fricke N., Conery A.L., Shen S., Pan W. (2018). A new class of synthetic retinoid antibiotics effective against bacterial persisters. Nature.

[37] Mohamed M.F., Abdelkhalek A., Seleem M.N. (2016). Evaluation of short synthetic antimicrobial peptides for treatment of drug-resistant and intracellular *Staphylococcus aureus*. Sci. Rep..

[38] Zhang Y., Yew W.W., Barer M.R. (2012). Targeting persisters for tuberculosis control. Antimicrob. Agents Chemother..

[39] Joers A., Kaldalu N., Tenson T. (2010). The frequency of persisters in Escherichia coli reflects the kinetics of awakening from dormancy. J. Bacteriol..

[40] Mandell J.B., Deslouches B., Montelaro R.C., Shanks R.M.Q., Doi Y., Urish K.L. (2017). Elimination of Antibiotic Resistant Surgical Implant Biofilms Using an Engineered Cationic Amphipathic Peptide WLBU2. Sci. Rep..

[41] Humphries R.M., Pollett S., Sakoulas G. (2013). A current perspective on daptomycin for the clinical microbiologist. Clin. Microbiol. Rev..

[42] CLSI (2016). Performance Standards for Antimicrobial Performance Standards for Antimicrobial Susceptibility Testing-26th Edition: CLSI Supplement M100S.

[43] Gallo S.W., Ferreira C.A.S., de Oliveira S.D. (2017). Combination of polymyxin B and meropenem eradicates persister cells from *Acinetobacter baumannii* strains in exponential growth. J. Med. Microbiol..

[44] Carpenter C.F., Chambers H.F. (2004). Daptomycin: Another novel agent for treating infections due to drug-resistant gram-positive pathogens. Clin. Infect. Dis..

[45] Feng J., Auwaerter P.G., Zhang Y. (2015). Drug Combinations against Borrelia burgdorferi Persisters In Vitro: Eradication Achieved by Using Daptomycin, Cefoperazone and Doxycycline. PLoS ONE.

[46] Pogliano J., Pogliano N., Silverman J.A. (2012). Daptomycin-mediated reorganization of membrane architecture causes mislocalization of essential cell division proteins. J. Bacteriol..

[47] Cue D., Junecko J.M., Lei M.G., Blevins J.S., Smeltzer M.S., Lee C.Y. (2015). SaeRS-Dependent Inhibition of Biofilm Formation in *Staphylococcus aureus* Newman. PLoS ONE.

[48] Barrett M.S., Jones R.N., Erwin M.E., Johnson D.M., Briggs B.M. (1991). Antimicrobial activity evaluations of two new quinolones, PD127391 (CI-960 and AM-1091) and PD131628. Diagn. Microbiol. Infect. Dis..

[49] Cohen M.A., Yoder S.L., Huband M.D., Roland G.E., Courtney C.L. (1995). In vitro and in vivo activities of clinafloxacin, CI-990 (PD 131112), and PD 138312 versus enterococci. Antimicrob. Agents Chemother..

[50] Balwan A., Nicolau D.P., Wungwattana M., Zuckerman J.B., Waters V. (2016). Clinafloxacin for Treatment of Burkholderia cenocepacia Infection in a Cystic Fibrosis Patient. Antimicrob. Agents Chemother..

[51] Levine D.P., Holley H.P., Eiseman I., Willcox P., Tack K. (2004). Clinafloxacin for the treatment of bacterial endocarditis. Clin. Infect. Dis..

[52] Yuan R.Y., Gour N., Dong X.Z., Jie F., Shi W.L., Zhang Y. (2019). Ranking of Major Classes of Antibiotics for Activity against Stationary Phase *Pseudomonas aeruginosa* and Identification of Clinafloxacin + Cefuroxime + Gentamicin Drug Combination that Eradicates Persistent *P. aeruginosa* Infection in a Murine Cystic Fibrosis Model. bioRxiv.

